# Community-based educational design for undergraduate medical education: a grounded theory study

**DOI:** 10.1186/s12909-019-1643-6

**Published:** 2019-07-11

**Authors:** Mora Claramita, Elsa Pudji Setiawati, Tri Nur Kristina, Ova Emilia, Cees van der Vleuten

**Affiliations:** 1 Department of Medical, Health Professions Education and Bioethics, Faculty of Medicine, Public Health, and Nursing, Universitas Gadjah Mada, Yogyakarta, Indonesia; 2 Department of Public Health, Faculty of Medicine, Universitas Padjajaran, Bandung, Indonesia; 3 Medical Education and Development Unit, Faculty of Medicine, Universitas Diponegoro, Semarang, Indonesia; 4 School of Health Professions Education, Maastricht University, Maastricht, The Netherlands

**Keywords:** Community-based education, Student-centered learning, Experiential-learning, Primary health care, General practice/family medicine

## Abstract

**Background:**

Community-based education (CBE) is strategically important to provide contextual learning for medical students. CBE is a priority for countries striving for better primary health care. However, the CBE literature provides little curriculum guidance to enhance undergraduate medical education with the primary health care context. We aim to develop a CBE framework for undergraduate medical education (from macro, meso, and micro curriculum levels) to engage students and teachers with better, more meaningful learning, within primary health care settings.

**Methods:**

We used a grounded theory methodology by interviewing eight medical educationalists and ten CBE teachers, followed with the coding process by sensitizing the concepts of ‘medical education’ and ‘primary care’, to explore any new concepts. The primary data originated from a developing country where the paradigm of high-quality primary health care is mostly unfamiliar. Three senior researchers from international associations of general practices from different countries provided validation to the results.

**Results:**

We identified a new framework for a community-based educational program. The micro-curriculum should offer opportunities for small group activities, ranging from simple to complex learning, emphasizing clinical skills, leadership, and teamwork to improve self-directed and collaborative practice. Sufficient role models and constructive feedback within primary care contexts are robust facilitators. For the meso-curriculum, comprehensive coordination on teacher-training and CBE program is needed. To ensure the sustainability of the program, faculty leaders and managers should include the macro-curriculum with a national postgraduate general practice curriculum and provide strong commitment.

**Conclusions:**

We designed a ‘CBE-tree’ model for the undergraduate medical curriculum. By using the CBE framework developed in this study, students and teachers may better comprehend the essential of primary health care.

## Background

### Contextualized learning in primary health care for medical education

Studies show that contextual learning or more meaningful learning for medical students can be enhanced by early exposure to community settings [1, 2]. Community-based education may promote socio-behavioral aspects of medical students in understanding factors affecting health problems in daily contexts [3, 4]. Factors other than diseases that influence the illness experiences include “the social determinants of health: the conditions in which people are born, grow, live, work, and age that affects health” [5]. To fully understand the influence of social determinants on individual patients, their family, and community, medical students should have sufficient socio-humanistic abilities. Fundamental principles for building socio-humanistic skills include effective communication and collaboration skills [6, 7].

There are essential skills for doctor-patient communication to train the health professionals, mainly listening skills and adequate observation skills, prior to developing proper and mutual informed and shared decision-making ability [6]. A recent study in the United Kingdom defined the topics taught in primary care medicine in undergraduate medical education as consulting and communication skills, leading and working in teams, and developing yourself; including novel topics such as learning disability, genetics, and multi-morbidity [8]. However, the community-based curriculum for undergraduate medical students in that study derived from the Royal College of General Practitioners (RCGP) postgraduate curriculum, where specialization in general practice has been existed and well-known for decades, in a western context. Some literature argues that engaging with general practices and family medicine specialists may lead to more profound reflections of learning for undergraduate medical students during intensive exposure to the community health problems [9]. However, not every country has a graduate general practice specialist program [9].

The World Health Organization/ WHO (2008) highly recommends all countries to strengthen their primary health care services to more accessible health care, through reformation of (1) universal coverage, (2) person-centered care, (3) public policy, and (4) leadership [10]. These four pillars underpin the high quality of primary care services and education.

### The importance of the community-based educational program and its problems

Using a proper CBE program design, medical students may be transformed into more sensitive and responsive future health professionals, who hopefully will have an interest, or at least acknowledge the valuable work at primary care settings. There are many medical schools around the world which implemented variations of CBE programs. Previous studies have confirmed the importance of CBE programs [11–15]. A study specifically demonstrated that community-based programs had been determined to be the most proper place for medical students to learn about health problems compared to hospital-based settings [16]. Generally, by early exposure to the CBE programs, medical students should be better in recognizing health problems in community settings [17].

However, the impact of the CBE program may be less than expected, mainly if there are a lack of teachers who could facilitate the students to reflect on primary health care contexts [18]. Literature suggests that a clinical educator is a clinician who is advance in clinical practice, enthusiastically ‘putting theories into medical education practice’, and involve in research-based services [19]. CBE teachers should have the same role. Nevertheless, in some countries, the doctors who work at primary care settings may be forced into being the general practice without proper preparation in their undergraduate medical education, have no graduate education specialist training in general practice, and limited exposure to systematic continuing medical education training in delivering better primary care services [9, 20]. Thus, in this kind of circumstances, many teachers involved in a ‘CBE program’ may not be able to inspire medical students to fully comprehend the importance of primary health care [9]. Related to the continuous incorrect general image of the primary health care services as “poor health care services, by poor health care workforce, to the poorest people” [9]; in this kind of situation, medical students may not learn better primary care services through a CBE program as intended.

### Curriculum and teaching strategies for a CBE program: lack of guidance

There is a generic framework of macro, meso, and micro curriculum levels for medical teachers in facilitating students’ learning [21]. At the micro-level, teachers should facilitate the learning process, i.e., by coaching, mentoring, evaluate, assess. At the meso-level teachers coordinate the teaching-learning program in different learning strategies and educational program. At the macro-level, leadership plays a vital role in the innovation in medical education, i.e., by the construction of competencies or professional abilities of the graduates to meet the local, regional, and global community needs. Knowledge, skills, and attitude are continuously framed within the three levels of the curriculum.

WHO specifically describes variations of activities of primary health care exposure for medical students and lessons learned from many countries, to approach the health needs of the people [22]. The other literature indicates the general objectives of a CBE program for developing countries [23]. Regarding the learning strategies, one study explains an instructional design explicitly, with lesson plans involving communication skills, which is an ability that is fundamental for community-based learning [24]. A recent study pointed out the use of ‘experiential learning cycles’ for instructional design of a CBE program, in rural clinical settings [25].

Each of those studies mentioned above, however, separately explaining about generic principles of teaching strategies in medical education but had not described the CBE program [21]; or, if the study had discussed about a CBE program, it had not touched a detailed instructional design [22–24]. One study described an instructional design principle but has a limited guide for teachers’ and students’ roles and tasks in each of the macro, meso, and micro curriculum levels of medical education [25]. Therefore, global guidance for medical teachers to do a proper CBE program in different levels of a curriculum remains unclear.

Considering the necessary framework of facilitating learning for a community-based educational program, we need a more detailed recommendation on systematic CBE learning characteristics for different levels of undergraduate medical curriculum (macro, meso, and micro levels). This study aims to develop a more systematic CBE instructional design for undergraduate medical education, to provide meaningful learning experiences for the students and faculty members, towards the benefit of the community.

## Methods

### Design

This study is a qualitative exploratory study with a grounded theory methodology approach [26–28]. We interview the subjects in this study and inductively coded the data by sensitizing the concepts of ‘student-centered learning’ [1–4], ‘curriculum levels’ [21], ‘experiential learning’ [29], as well as principles of ‘primary health care’ and ‘general practice’ or ‘family medicine’ [9, 20], until saturation.

### Subjects

We purposively selected the participants based on their intensive studies of community-based medical education programs (Table 1). Eight medical educationalists (Doctorate or Master Degrees), from five most reputable medical schools in Indonesia, participated in the interviews. They were from a developing country where primary health care concepts were mainly oriented to the curative-care rather than investing in the prevention and continuity of care [30]. Although a universal coverage insurance system was recently established in this country [20, 30], the graduate general practice specialist education does not yet exist [20].

**Table 1:** Backgrounds of community-based educational researches/publications of medical educationalists as participants in this study

Medical Educationalists	Researches/Publications
1	Report of the Centre for Community Health Care (CCHC) involving all primary health care centers in Yogyakarta Special Province and all medical teachers within the faculty from 1980-1990. Faculty of Medicine Universitas Gadjah Mada - supported by the Rockefeller Foundation US. Report of a Community and Family Health Care Program with Inter-Professional Education (CFHC-IPE) involving medical, nursing and dietician students of Universitas Gadjah Mada, primary health care centers and general practitioners in Yogyakarta Special Province.
2	A dissertation of generic objectives for community-based education in undergraduate medical program: The perspective from developing countries: 2000-2005. Faculty of Medicine Universitas Diponegoro - funded by QUE Project of Indonesia.
3	A dissertation of attachment of medical students learning clinical skills to primary health care centers to prepare for clerkship: 2008-2011. Faculty of Medicine Universitas Gadjah Mada - funded by NPT Project The Netherlands.
4	A thesis of elderly attachment to clinical skills training (the ‘healthy-Saturday’). 2012. Integrated patient management program. Involving Yogyakarta Elderly Association under the Provincial Office of Yogyakarta, family doctors of North Yogyakarta and all medical students of Faculty of Medicine Universitas Gadjah Mada Year 2 from 2007-2012.
5	A national presentation on the first 1000 days of life (one student – one baby). A community-based attachment to medical students at Faculty of Medicine University of Hasanuddin Makassar at the Ministry of Research, Technology and Higher Education. 2014. http://unhas.ac.id/1000harikehidupan/
6	A presentation during a workshop on leadership in primary health care women medical teachers. Asia-Pacific Family Medicine Conference in Jeju South Korea. 2012. Presented activities of primary health care exposures to medical students. Involving primary school children at North Sumatra and clerkships students at University of North Sumatra (USU) Medan, from 2005 – at present.
7	A report of community-based medical education. Universitas Airlangga, Surabaya. 2011. Involving primary schools for disabled students in East Java and medical students in Year 3 from 2007 – at present.
8	A report of community-based medical education. Universitas Mulawarman, Samarinda. 2011. Involving all Primary Health Centers in the district and medical students in final years from 2008 – at present.

For a triangulation, we used different data sources by interviewing ten medical teachers from the same country, who actively coached students within CBE settings in the last 5 years. Seven of them are active medical doctors who work in primary care settings and general practitioners (without a formal graduate education in general practice). Two of them were health professionals other than physicians and one hospital specialist. To increase the validity of the findings, we asked international researchers who were also active general practitioners and senior leaders of CBE programs in their countries, from Egypt, India, and Belgium. The international experts were coming from different countries and continents, which may have better primary health care systems and the graduate general practice education already established for many years.

### Instruments

Two questions guided the interview in this study: (1) What are the essential CBE learning characteristics that you think can be helpful to enhance the comprehension of primary health care of medical students? (2) What are your further recommendations to make the CBE learning characteristics more systematic to each level of undergraduate medical students? To the international experts we asked: (1) Do you agree/not agree with the framework as the results of the interviews. (2) What do you recommend further?

### Analysis

Recordings from the interviews were: 1) transcribed, 2) categorized, 3) coded, 4) constantly-compared, 5) continued to find the emergent themes, and 6) finally thematically interpreted based on the process of grounded theory methodology [26–28]. Three coders with different academic backgrounds (MC-medical education, FM-family medicine, DH-anthropology) categorized the transcripts individually and regularly met for discussion. In the first two meetings, the coders listed 16–22 issues concerning CBE learning characteristics and then grouped the words such as socio-behavioral, anthropology, illness, culture, communication, into “community orientation.” Other words such as feedback, role model, leadership, and instructors grouped into “role of teachers.” The coding process was continues iteratively for 6 weeks, aiming towards an agreement between coders on the selection of categories.

Categories were carefully discussed and ultimately changed into specific terms, e.g., “guidance,” “primary health care context,” and “learning opportunities,” in the final framework. During the third and fourth week, the coders continued to see connections between categories. The coders used the ‘coding paradigm’, which involved constantly-comparing the findings by the dialectical process [26–28]. The three coders with a variety of scientific-subject backgrounds argued thoughtfully. Negative data was cautiously analyzed and discussed. Ultimately, coders discovered that their different views based on their diverse educational backgrounds might be complementary and serve to strengthen the findings of this study. In the fifth meeting, the three coders agreed that the data and coding process was saturated. The coders, together with the authors in this study, then interpreted the findings by constructing the meaning of the results into emergent themes. Through this process of ‘grounded’ interpretation, we identified a framework for more systematic training of community-based education for medical students through macro, meso, and micro curriculum aspects.

## Results

**Fig. 1:**
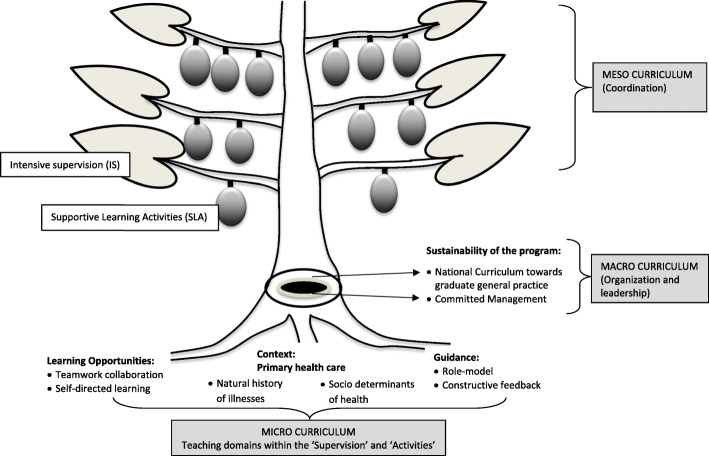
The framework of community-based education for medical students illustrated as a ‘CBE-Tree’ towards better comprehension of primary health care

The result of this study is a framework of more systematic training of community-based education for medical students illustrated as a ‘CBE-tree’ (Fig. 1). The participants in this study and their educational and research backgrounds explained in Table 1. As leaders, the participants have been involved scientifically and practically in a CBE program in their institutions. The CBE learning characteristics described in the ‘CBE-tree’ model as the ‘roots’ of the tree and include elements of the micro-curriculum: a) Opportunities for the students to enhance self-directed learning and teamwork collaboration, b) Guided by allowing adequate participation with good role models and constructive feedback to stimulate reflection from the teachers, and c) A more contextual learning that emphasizes both ‘general medical content’ (the preventions across natural history of illnesses) and primary care medicine/family medicine principles (person-centered care, continuity of care, holistic care, and comprehensive care). These micro-components will be transported-up inside the ‘Supported Learning Activities’ (SLA) and ‘Intensive Supervision’ (IS) described in this study as the ‘fruits’ and ‘leaves’ of the ‘CBE-tree,’ in the meso-curriculum coordination. We illustrated the order of knowledge-skills-attitude acquisitions of the students from simple to complex learning: the more supervision (‘leaves’) and fewer activities (‘fruits) for the lower; and reversely for the higher levels of students, to approach the self-directed learning principles. Clear direction of the macro-curriculum on graduate ‘general practice’ and ‘committed management’ at both national and faculty levels; which described as the ‘bark’ and ‘trunk’ of the ‘CBE-tree,’ will sustain these intended principles of CBE learning. The macro levels will provide maximum protection to ensure the continued existence of the CBE program. Table 2 explains the detail of Fig. 1. Guidelines for teachers to coordinate the CBE meso-curriculum, are presented in Tables 3 and 4.

**Table 2:** The CBE learning design for undergraduate medical curriculum based on the ‘CBE-tree’ in this study

Principles		Illustrations (Shows by Figure 1)	Quotations
Micro-curriculum	Students’ learning strategies:	Root	
	1. Self-directed learning	Root	*“Learning in community settings may capture many experiences. We should stimulate students to reflect on their experiences by using written diary of audio-visual records and use them to plan their future learning.”*
	2. Teamwork collaboration	Root	“*Students will work together during explorations and discussion sessions with the community. Although students should mastering the abilities individually, in the process, they need each other to get peers feedback, to do complimentary tasks and to learn from other health professionals.”*
	Teachers’ facilitation strategies:	Root	
	3. Role model	Root	*“Role model of the teacher is certainly needed. Teachers should show ‘passion’, so that students can feel it more than via direct feedback. I call this: a leadership by example.”*
	4. Constructive Feedback	Root	*“Good feedback would really stimulate students to think and learn by themselves and so the role of the teachers is “Tut Wuri Handayani” or from behind we empower, as stated by the first Minister of Education of Indonesia in early 1950s.”*
	Contents to be facilitated:	Root	
	5. Medical content (emphasizing of 5 levels of prevention – natural history of illnesses)	Root	“*The 5 levels of prevention emphasizing the natural history of illnesses are important to be understood by future doctors. When they meet a patient, besides exploring on patients intention to visit, doctors should also aware of risks detection of priority illnesses on particular age group and how to prevent risks to move further in the levels of prevention.”*
	6. Socio-determinants of health	Root	“*The concept of diseases and illness perceptions should be understood by medical students as well as socio-cultural values of our society. To involve the people during learning with them in trying to overcome health problems are the challenge.”*
Meso-curriculum	Coordination and training of simple to complex levels of learning:	Branches	*“An environment of closer knowing and care towards the people should begin since early medical education at community settings and continue throughout their study.”* *“Medical students may start to learn with the healthy people surrounding them, in the family, neighbourhood, community, and realize that every person has risk-exposures of priority illnesses. In more advances years, students may work in local clinics to start to help manage the patients’ problems when they already have presenting symptoms or maybe diagnosed.”*
	7. Supportive learning activities	Fruits	*) More about supportive learning activities are described in Table 3
	8. Intensive supervision	Leaves	*) More about intensive supervisions are described in Table 4
Macro-curriculum	Sustainability of the program:	Trunk	
	9. Commitment of the management: National to faculty levels	Trunk	*“We need somebody to state the need of early exposure of primary health care at community settings in the national standard of medical doctors’ competencies and we must have a graduate program on primary care medicine, so it will give all faculty of medicines clearer direction.”*
	10. National curriculum towards graduate general practice	Bark	*“This primary health care exposures program needs planning, organizing, actuating, monitoring and good evaluation. The management support is crucial. To prioritize this program is highly necessary.”* *“This kind of learning activities should be created by integrated departments and not a stand-alone program.”*

**Table 3:** The recommended meso-curriculum namely “Supported Learning Activities” within a systematic CBE framework in the ‘CBE-tree’ in this study

“Supportive Learning Activities” in which teachers should coordinate to the micro-content:	Examples of “Supportive Learning Activities”		Basic micro abilities to be enhanced	Topics (Gradual)
1. Small Group work (2 to 5 students) 2. Learning strategy: inductive (starting from exploration and the conclusion or intervention comes later in the later stage) 3. Tasks: a. To interact with people b. To do unobtrusive observation c. To reflect on experiences • Using log-book or portfolio • Adequate feedback on listening skills, observation skills, reflection-planning, two-way shared decision making skills d. Continued tasks – periodic e. The tasks should match with block-theme f. Gradual tasks 4. Settings: a. Field work b. Gradual clinical settings c. Gradual focus on individual/ family/ community 5. Proper period of time for learning cycle: field work activities ➔ learning process ➔ feedback ➔ learning plan 6. Assessment: a. Continuous constructive feedback b. Observation-based assessment	Year 1	1. Tutorial discussions, mini lectures with cases 2. Survey with guided questions based on block-topics 3. Learning ‘symptom and sign’ in daily settings: a. Laboratory settings: Role-Play, Simulated Patient b. Field work of VS at community settings (with log book and feedback session) 4. Individual unobtrusive observation-participation a. Field work of observation and interaction with common people and their daily activities (farmers, fisherman, executives, micro economic sellers, teachers, etc.) b. Field work of observation and interaction with specific group of people and their daily activities (disabled, HIV, etc.)	Listening skills Observation skills Reflection skills	Individual as unit of care
	Year 2	Learning risk factors, social determinants of health, symptom and sign in daily settings: a. Laboratory settings: Role-Play, Simulated Patient b. Field work of VS at community settings (with log book and feedback session) to various age group	Listening skills Observation skills Reflection skills	Family as unit of care
	Year 3	Learning Early Detection of Natural History of Illness, High Risk, Priority Illness, Chronic Illness and Clinical education in various settings (link to community settings): a. Community based settings: Individual unobtrusive observation-participation: Field work of observation and interaction with specific group of people and their daily activities (disabled, HIV, etc.) b. Primary health care c. Hospitals – outpatient clinics, home care, home visits	Listening skills Observation skills Reflection skills Two-way shared decision making skills Integrated clinical skills in primary health care	Special Age group as unit of care
	Clinical years	Learning of : a. Diagnosis for Individual (Clinical-Sub Clinical-High Risk) b. Diagnosis for Family health problems c. Diagnosis for Community health problems d. Patient education using two-way interaction and shared clinical decision making e. Community education- to communicate effectively with the community member and/or with key person including on how to consider the sociocultural aspects	Listening skills Observation skills Reflection skills Two-way shared decision making skills Integrated patient management	Community as unit of care

**Table 4:**
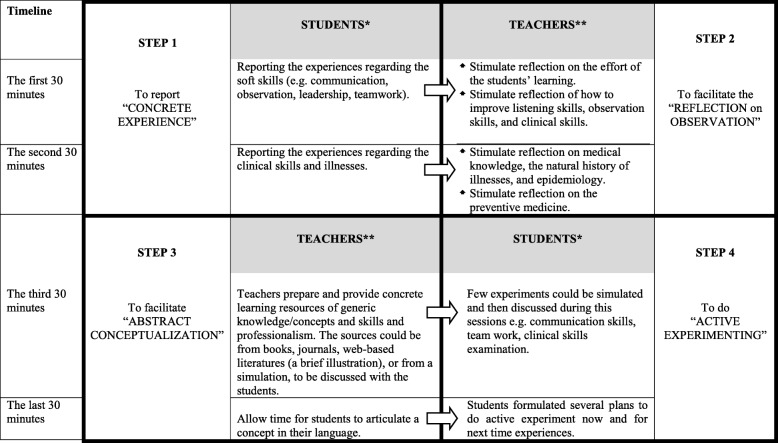
The recommended meso-curriculum namely “Intensive Supervision” of a CBE framework in this study: the feedback sessions - based on the ‘experiential learning cycle’ by Kolb (2010)

*) Students: Preferably small group of less than 5 students caring for one family; **) Teachers: preferably general practitioners from graduate training of family medicine specialists/ general practice

During the member-checking process, participants in this study emphasized some elements on feedback guidelines, as presented in Table 4. The participant highlighted the importance aspects of feedback (part of the micro-curriculum), which should be delivered using concrete steps related to experiences that students already had at the community settings (part of the meso-curriculum). We used the ‘experiential learning cycle’ from Kolb to create a feedback guide for the CBE context [29].

The international experts commented that the overall ‘CBE-tree’ model is a simple visual illustration of a framework of a more systematic CBE. The strength relies on its intensive levels of exposures of social determinants of health, emphasizing continuous preventions across the life span of all genders and ages, and the use of different kinds of learning strategies ranging from small group discussions to role-play activities in classes, as well as internships at community health centers and primary health care settings. The main suggestion from an expert was to try to reflect on a ‘WONCA-tree’ figure of graduate general practice/family medicine since the general design and medical scope are closely interrelated [31].

## Discussions

We describe a theoretical framework to design and implement a CBE program in a more systematic and meaningful way, of approaching the socio-accountability of medical institutions. By using the ‘CBE-tree’ framework in this study, lessons learned may be significantly more beneficial for the students, faculty members, and participating community. The challenge of bringing the educational issues into implemented training is illustrated by bringing the idealism of the original concepts of CBE (presented as the micro-curriculum of the ‘CBE-tree’) up to coordinating the implementation (presented as the meso-curriculum of the ‘CBE-tree’). Whereas, the most significant challenges are to have the macro-curriculum of a national graduate curriculum of general practice aimed towards strengthening primary health care and the commitment of faculty managers to allocate and prioritize the budget, resources and time for the undergraduate CBE program.

In this study, one of the elements of the micro-curriculum should provide opportunities for students to enhance self-directed and collaborative learning. Confirming these findings, medical education in the twenty-first century should move towards a model of more community-oriented care. Contextual-learning should be encouraged, possibly with other health professions’ students, to early introduce the concept of interprofessional collaborative (IPC) practice [1–4, 7, 9, 32, 33].

As described in one of the micro-curriculum elements, the context of the CBE learning program is closely related to general practice or family medicine as described in the ‘WONCA-tree’ model [31]. However, the aim and the details are much different between the two ‘tree-models’. The ‘WONCA-tree’ model emphasizes the holistic principles of family medicine in caring for the patients, while our ‘CBE- tree’ model focuses on the framework of instructional design of a community-based education program for undergraduate medical students. The connection between the ‘WONCA-tree’ model and our ‘CBE-tree’ model, in the implementation, relies on the content of the feedback on primary health care and teachers who deliver the feedback. Students need proper guidance by inspiring role models who deliver constructive feedback to assist students’ reflection on CBE experiences. It is likely that the doctors who work at the primary care settings, who more fully understand the principles of family medicine, will be the most suitable role models and mentors for the students [9]. Those teachers are also expected to be the general practitioners or family medicine specialists by formal graduate training through which they have sufficient knowledge of practicing primary health care services.

It is evident that any effort to introduce primary health care in any country by implementing CBE programs in many medical schools may be unattractive or misinterpreted by those who participate, primarily if primary health care still viewed as the ‘second class’ of care [9, 20, 34]. In some countries, the career pathway of doctors working at primary health care levels is set by ‘destiny’ and not by choice of destination [9]. Nevertheless, when we engage in community-based education, there is no other option than to prepare the students to be the agents of social change [32]. In this regard, to be the future ‘change-agents’, students need to fully comprehend the essentials of primary health care since the beginning of their medical education, by reflecting on their experiences in a CBE program and role modeling their teachers from primary care settings. Students should learn in the context of the epidemiological transition towards more chronic conditions and multimorbidity, where a paradigm-shift from disease-oriented care towards goal-oriented care is of utmost importance [33]. Essentially, person-centered care and continuity of care are the keys for excellent health care services, combined with interprofessional collaborative practice to provide comprehensive care for the patients oriented towards patient-safety [6–10]. With this purpose, general practice or family medicine graduates can help medical students to better comprehend these shifting paradigms in delivering high quality of health care [9, 20, 34].

The meso-curriculum needs qualified teachers who could coordinate a variety of learning experiences [21]. In this study, we envisioned the meso-curriculum involving gradual progress while the students move to later years of medical education. Teachers should be able to coordinate all learning and teaching opportunities, as described in the tables. Support for teachers to provide more constructive feedback is necessary. A guideline for conducting a feedback session with the students based on the ‘experiential learning cycle’ in this study could be introduced in continuous faculty development programs. By using this guideline, contextual learning can become more meaningful for the students, teachers, and ultimately participative community members.

Another backbone for the success of community-based educational programs, as illustrated in the macro-curriculum in this study, is faculty commitment towards the program. The responsibility could be shown by time allocation for the CBE program and should not interfere with other class-based educational activities such as laboratory practical sessions, lectures, and tutorial sessions. An excellent example, there is one university which designs the first few orientation-day of their medical students are scheduled to be at the primary care settings with their primary care instructors, instead of being at the faculty of medicine to meet the deans and lecturers. Therefore, students get along earlier with the primary care doctors, other health professionals, and a family whom he/she will be attached to, for the rest of their medical education (Table 1, as explained by respondent No.5). In this example, assessing and responding to any problems of the assigned family (called ‘partner-family’), were also prioritized together with any classes’ activities. As a result of the high commitment of faculty managers towards the CBE program in that study, the maternal-mortality rate in that community working with the particular faculty of medicine of eastern Indonesia was reported zero for the last 3 years.

Additionally, the faculty managers should show their commitment by providing all committed resources for the CBE programs, including hospital staffs, community medical center doctors, nurses and midwives, community leaders, even the private company/stakeholders nearby the university to be fully supportive and maximally available to be consulted by the students, at any time using any mode of communication. In this arrangement, managers will automatically prioritize the budget, and human resources for the CBE program; including the schedule of the CBE program in the overall curriculum. Faculty development and regular training for teachers should also be maintained. Without a full commitment from the faculty managers, a CBE program usually acts as a stand-alone from the curriculum and is led by only a small number of committed staff.

Future study should test the implementation of the CBE framework for undergraduate students in this study. We should also learn from students’ perceptions and furthermore from teachers as well as community members. The design may not completely answer the needs for an ideal CBE program, however many of the themes were found to be essential elements in current theories applied in experiential-learning. Further reinforcing is the insightful statements from the international experts concerning the importance of community-based education, to meet the ongoing global efforts of strengthening primary health care [34].

## Conclusions

We propose the use of a community-based educational framework in this study (the ‘CBE-tree’ model) for undergraduate medical education. By using the ‘CBE-tree’ model, we hope to approach a more meaningful learning for students, staff, and community members towards a strong primary health care services and education.

## Data Availability

Data of this qualitative study were obtained from interviews (with the participants) and coding processes (by the research assistants) and were in Indonesian language. Data for validation of the CBE framework were obtained via electronic mail from international experts and were in English. The data could be retrieved on reasonable request.

## Abbreviation

CBE: Community-based education
IS: Intensive supervision
RCGP: Royal College of General Practitioners
SLA: Supported learning activities
WHO: World Health Organization
WONCA: WONCA-World Organization of Family Doctors/ General Practitioners (WONCA has been regarded as one term and not anymore an abbreviation)
